# Multivariate analysis of causal factors influencing accuracy of guided implant surgery for partial edentulism: a retrospective clinical study

**DOI:** 10.1186/s40729-021-00313-2

**Published:** 2021-04-19

**Authors:** Atsushi Matsumura, Tamaki Nakano, Shinji Ono, Akihiro Kaminaka, Hirofumi Yatani, Daijiro Kabata

**Affiliations:** 1 Department of Fixed Prosthodontics, Osaka University Graduate School of Dentistry, 1-8 Yamadaoka, Suita, Osaka, 565-0871 Japan; 2 Department of Medical Statistics, Osaka City University Graduate School of Medicine, 1-4-3 Asahi, Abeno-ku, Osaka, 545-8585 Japan

**Keywords:** Computer-guided surgery, Surgical guides, Partial edentulism, Multivariate analysis

## Abstract

**Abstract:**

**Background:**

In dental implant treatment, the placement position of the implant body is important. The hypothesis is that there are factors that have a greater impact than the factors that have been studied so far.

**Material and Methods:**

The deviation between planned and actually placed implants was measured three-dimensionally by modified treatment evaluation method in 110 patients who underwent implant placement with guided surgery for partial edentulism. Ten factors that seemed to affect errors in placement were selected: the type of tooth, type of edentulism, distance from the remaining teeth, the type of implant, implant length, number of implants, method of guidance, the number of teeth supporting the surgical guide, number of anchor pins, and presence or absence of a reinforcement structure. The effect of each factor that corrected each confounding was calculated using multivariate analysis.

**Results:**

In this study, 188 implant bodies were set to target, and the errors measurement data of the implant position were as follows: average Angle, 2.5 ± 1.6° (95% CI 2.25–2.69); Base, 0.67 ± 0.37 mm (95% CI 0.62–0.72); and Apex, 0.92 ± 0.47 mm (95% CI 0.86–0.98). As the result of multivariate analysis, larger errors were present in the partially guided group than the fully guided group. The number of teeth supporting the surgical guide significantly influenced the error in placement position. The error caused by the number of anchor pins was significantly different for the Angle. Similarly, the presence of the reinforcement structure influenced the error significantly for the Angle.

**Conclusions:**

It was suggested that the smaller errors could be present by performing guided surgery with full guidance and devising the design of the guide such as the number of teeth supporting the surgical guide, the setting of the anchor pin, and the reinforcement structure.

## Background

In dental implant treatment, the placement position of the implant body is important to produce an esthetically pleasing prosthesis [1–4], and an inappropriate placement position is considered to be a risk factor for peri-implantitis [5–7]. The frequency of use of surgical guides has increased because guided surgery is believed to allow more accurate placement than free-hand surgery [8–10], and many studies have focused on the accuracy of guided surgery [11, 12]. According to Tahmaseb et al. [13], the error during guided surgery is 1.2 mm at the center of the platform of the implant body, 1.4 mm at the apex, and 3.5° at the angle. These findings indicate that guided surgery has clinically satisfactory accuracy.

With respect to factors that cause errors in the placement position during guided surgery, such errors are reportedly larger in the molars than in the anterior teeth [14, 15], in cases involving implants in free-end defects than in intermediate defects [16], and in cases involving implants with longer than shorter bodies [17, 18]. Furthermore, the error is reportedly larger in partially guided than fully guided surgery [19, 20]. However, these studies treated each factor as a univariate factor, resulting in the inability to consider the influences of confounding factors on the results.

Past reports suggest that the movement during drilling when using surgical guides affects the error of the placement position [21, 22] and that the error of the placement position can be decreased by setting anchor pins [23, 24]. In reality, however, few reports have focused on surgical guide designs, such as the number of teeth supporting the surgical guide, number of anchor pins, and the presence or absence of a reinforcement structure.

Based on the above information, the present study was performed to clarify the factors that affect errors of implant placement position in implant guided surgery. For this purpose, the confounding between each factor was corrected using a multivariate analysis. The magnitude of the impact was then calculated, and how clinicians should devise to reduce the error of guided surgery was statistically examined.

## Materials and methods

### Study design and population

This study was designed as a retrospective study and participants were 122 patients who underwent implant installation with guided surgery from 1 September 2015 to 31 May 2018 at the Osaka University Dental Hospital and approved by the Department of Dentistry and the Hospital Ethics Committee of the Faculty of Dentistry of the Osaka University Graduate School of Dentistry (approval No. H29-E45).

This trial is reported in accordance with the STROBE (STrengthening the Reporting of OBservational studies in Epidemiology) statement (https://www.strobe-statement.org/) for improving the quality of reporting of observational studies.

The inclusion criteria are patients aged 20 years or older, who are not a general contraindication to oral surgery, and who have 6 or more teeth remaining. The clinicians used surgical guides based on model scan data on digital treatment planning software (Nobel Clinician; Nobel Biocare, Kloten, Switzerland) and implants produced by Nobel Biocare. The method of matching the scanned model and CBCT data called Smart Fusion is applied by the manufacturer’s rule that there are 6 or more remaining teeth. The exclusion criteria were patients who have remaining teeth with significant mobility, having discontinued the use of the surgical guide during drilling, and patients who received tissue graft or bone graft at the implant placement. Twelve patients were excluded because they met the exclusion criteria. In total, 110 patients (39 men, 71 women; mean age, 55.1 ± 15.5 years; 188 implants) who met the inclusion criteria were included in this study.

Implant installation was performed by 23 dentists from the Department of Fixed Prosthodontics, and all had > 5 years of experience with guided implant surgery. All procedures were performed according to the Nobel Biocare protocol.

In this study, when a CBCT scan after surgery is performed, a subject has to attach a guide used in surgery. Therefore, the bite index made by silicon patty was prepared to stabilize the position of the guide at the CBCT scan.

### CBCT scan

The CBCT apparatus used in this study was the Alphard 3030 (Asahi Roentgen Kogyo Co., Ltd., Kyoto, Japan), and the imaging conditions were set as shown in Table 1. The patient maintained a sitting posture at the time of imaging. CBCT scans were performed twice, pre-operation and post-operation. Immediately after the implant installation, the surgical guide used for the surgery was attached to the patient and a CBCT scan was performed with the bite index prepared in advance to stabilize the position.

**Table 1:** Cone-beam computed tomography imaging parameters

Field of view	Diameter: 102 mm
	Height: 102 mm
Voxel size	0.2 mm
Tube voltage	80 kVp
Tube current	7 mA
Exposure time	17 s

### Implant validation

The obtained CBCT imaging data were constructed three-dimensionally using digital image measurement software (coDiagnostiX; Dental Wings, Montreal, Canada). The deviation measurement of the three-dimensional jaw bone model was also performed on the same software.

**Fig. 1:**
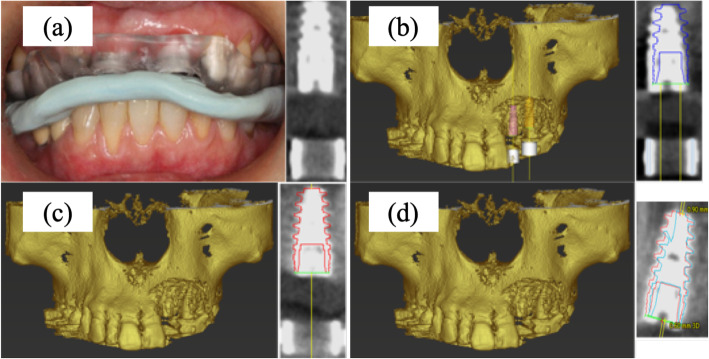
Evaluation method of implant validation. **a** Immediately after the implant installation, the surgical guide used for the surgery was attached, and a cone-beam computed tomography scan was performed with the bite index prepared in advance to stabilize the position. **b** The position of the planned implant body was specified from the position of the metal sleeve in the jaw bone model, and implant model 1 was installed there. **c** Implant model 2 was placed according to the implant body actually inserted. **d** The deviation between the implant models was measured three-dimensionally on the jaw bone model

**Fig. 2:**
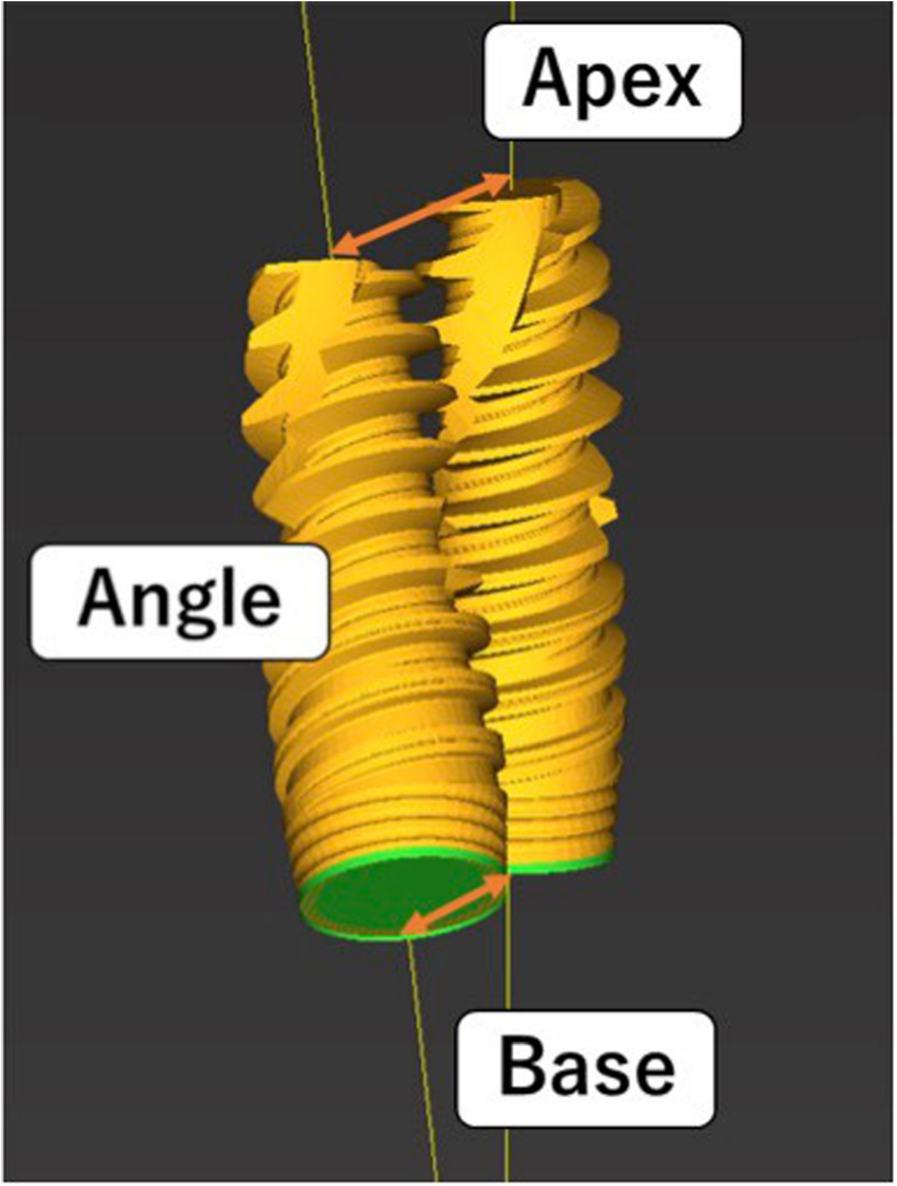
Measurement outcomes. Angle (°): angle between the long axes of the implant model. Base (mm): distance between the platform centers of the implant model. Apex (mm): distance between the implant model tips

The modified treatment evaluation method was as follows (Fig. 1). Three-dimensional construction was performed based on Digital Imaging and Communications in Medicine data obtained by the CBCT scan, and a three-dimensional jaw bone model was produced. Because the CBCT scan was performed with the surgical guide used for the surgery, the obtained data could be used to delineate the metal sleeve contained in the surgical guide. Based on the positional relationship specified at the time of surgical guide manufacture, the position of the planned implant body was specified from the position of the metal sleeve in the jaw bone model, and implant model 1 (IM1) was installed at the position. Then, implant model 2 (IM2) was placed according to the implant body actually inserted. The deviation between the two IMs was measured three-dimensionally on the jaw bone model, and this was defined as the error of the implant placement position in this experiment. The measurement outcomes were the angle between the long axes of the IM (Angle (°)), the distance between the platform centers of the IM (Base (mm)), and the distance between the IM tips (Apex (mm)) (Fig. 2). Finally, the intraclass correlation coefficient (ICC) was calculated and the intraexaminer and interexaminer reliability were assessed.

### Factors affecting the error of the placement position

Based on past reports [14–20, 23–28] and clinical experience, 10 factors were selected as those that affect the error of the placement position. Three factors were selected as missing teeth-derived factors: the type of tooth, type of edentulism, and distance from the remaining teeth. Four factors were selected as implant-derived factors: the type of implant, implant length, number of implants, and method of guidance. Finally, three factors were selected as guide design-derived factors; the number of teeth supporting the surgical guide, number of anchor pins, and presence or absence of a reinforcement structure.

**Fig. 3:**
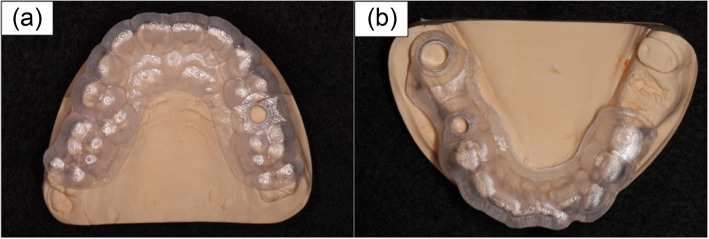
Example of missing teeth-derived factors. **a** Tooth type: premolar, types of edentulism: intermediate defect, and the distance from the remaining tooth to the placement position: 1 tooth. **b** Tooth type: premolar and molar, types of edentulism: free-end defects, and the distance from the remaining tooth to the placement position: 1 tooth and 3 teeth

Tooth types were classified into three groups (incisors, premolars, and molars) and two types of edentulism (intermediate defects and free-end defects). The distance from the remaining tooth to the placement position was expressed by how many teeth were separated from the adjacent teeth to the placement site (Fig. 3).

**Fig. 4:**
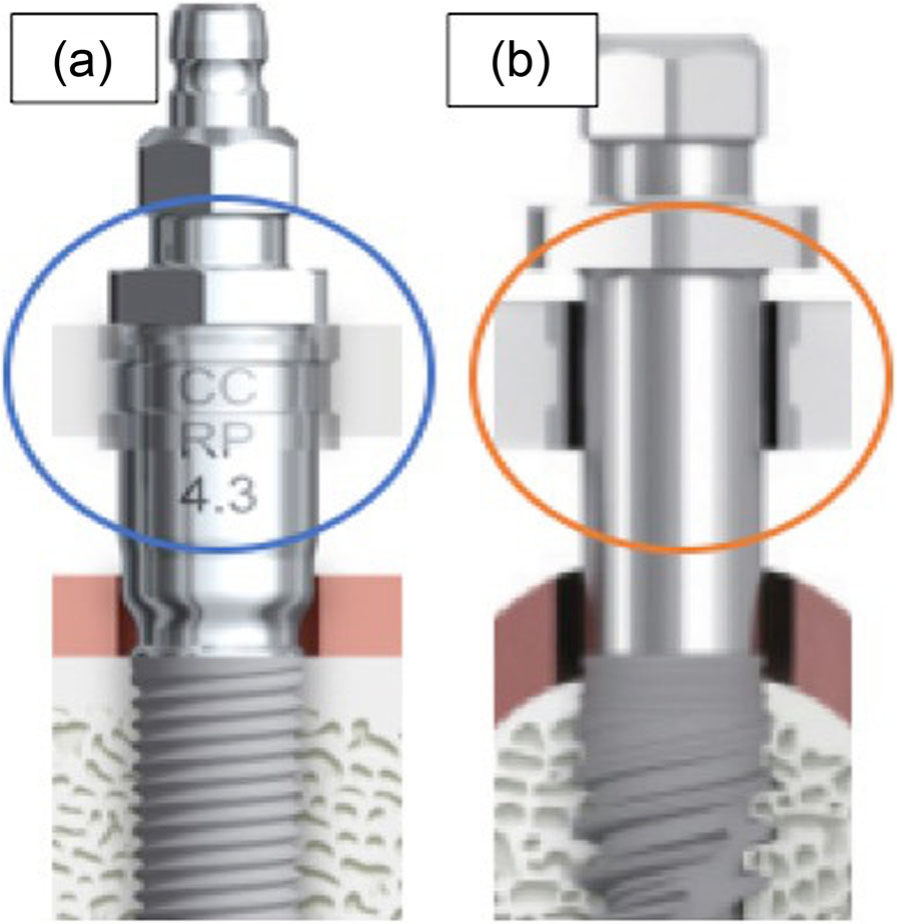
The types of implants were classified into groups with large and small differences in the diameter between the implant mount and the metal sleeve in the surgical guide. **a** The smaller gap group contains NobelReplace® CC implants, NobelParallel™ CC implants, and NobelReplace® Tapered implants. **b** The larger gap group contains NobelActive™ implants.

The types of implants were classified into groups with large and small differences in the diameter between the implant mount and the metal sleeve in the surgical guide (Fig. 4). The smaller gap group contains NobelReplace® CC implants, NobelParallel™ CC implants, and NobelReplace® Tapered implants. The larger gap group contains NobelActive™ implants. All these implants were produced by Nobel Biocare.

The method of guidance was classified into two groups: use of the surgical guide until implant placement without free-hand depth adjustment after placement (fully guided group) and use of the surgical guide until implant placement with subsequent free-hand depth adjustment or until the final drilling and free-hand placement (partially guided group).

The anchor pins were metal pins with a diameter of 1.5 mm inserted into the jaw bone for fixation of the surgical guide, and the factor was the number of anchor pins set in the surgical guide.

**Fig. 5:**
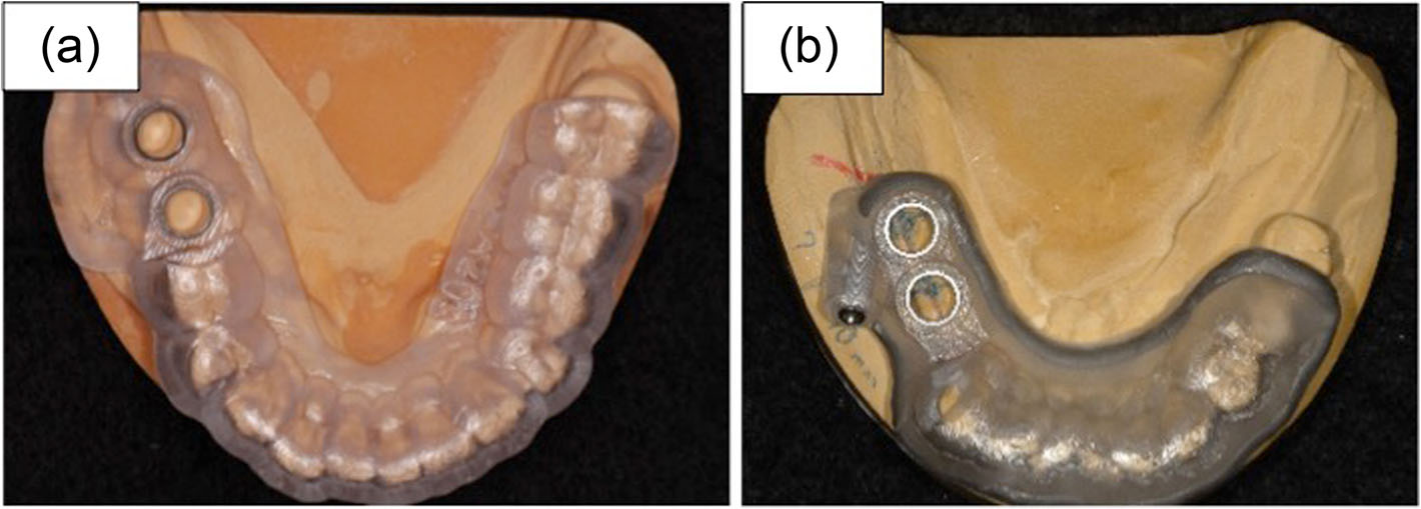
Example of guide design-derived factors. **a** The number of teeth supporting the surgical guide: 12 teeth, number of anchor pins: 0, and presence or absence of a reinforcement structure: absence. **b** The number of teeth supporting the surgical guide: 9 teeth, number of anchor pins: 1, and presence or absence of a reinforcement structure: presence

The reinforcement structure was cobalt-chromium alloy with 1.1-mm thickness and 2.8-mm width. The alloy was added around the surgical guide to prevent fracture and deflection of the surgical guide, and the patients were classified into those with and without a reinforced structure (Fig. 5).

### Statistical analysis

In order to examine the effects of the above 10 variables described in the previous paragraph on each outcome variable indicating the error in the placement position, Angle, Base, and Apex, the multivariable nonlinear regression analyses were performed with each outcome variable as a function of the 10 risk factor candidates. Correspondence between data obtained from the same case was corrected using the Huber–White robust covariance sandwich estimating method. Hypothesis testing was performed using a 5% two-sided significance level. All analyses were performed using the R version 3.5.1 and rms packages [29].

## Results

The intraexaminer and interexaminer reliability were sufficiently high (ICC ≥ 0.75).

The error measurement data of the implant position for 188 implant bodies were as follows: average Angle, 2.5 ± 1.6 ° (95% CI 2.25–2.69); Base, 0.67 ± 0.37 mm (95% CI 0.62–0.72); and Apex, 0.92 ± 0.47 mm (95% CI 0.86–0.98). The average number of implant bodies per patient was 1.71 (Table 2).

**Table 2:** Implant validation

	Angle (°)	Base (mm)	Apex (mm)
Average (95% CI)	2.5 ± 1.6 (2.25–2.69)	0.67 ± 0.37 (0.62–0.72)	0.92 ± 0.47 (0.86–0.98)
Median	2.30	0.61	0.78
Minimum	0.00	0.08	0.08
Maximum	7.90	2.17	2.40

Total implants: 188 implants

Average number of implants per patient: 1.71 implants

*CI* confidence interval

The baseline data of each explanatory variable are shown in Tables 3, 4, and 5. Table 6 shows the influence of each factor on the error of the placement position.

**Table 3:** Baseline data of explanatory variables

	Explanatory variables		*n*	Percentage
Missing teeth-derived factors	Type of tooth	Incisors	21	11.2%
		Premolars	67	35.6%
		Molars	100	53.2%
	Type of edentulism	Intermediate defects	98	52.1%
		Free-end defects	90	47.9%
	Distance from remaining teeth	1 tooth	148	78.8%
		2 teeth	22	11.7%
		3 teeth	15	7.9%
		4 teeth	3	1.6%

**Table 4:** Baseline data of explanatory variables

	Explanatory variable		*n*	Percentage
Implant-derived factors	Type of implant	Large difference in diameter	58	30.9%
		Small difference in diameter	130	69.1%
	Implant length	7.0 mm	11	5.8%
		8.0 mm	24	12.8%
		8.5 mm	33	17.6%
		10.0 mm	82	43.6%
		11.5 mm	28	14.9%
		13.0 mm	9	4.8%
		15.0 mm	1	0.5%
	Number of implants	1 implant	54	28.7%
		2 implants	105	55.9%
		3 implants	21	11.2%
		4 implants	8	4.2%
	Method of guidance	Fully guided	141	75.0%
		Partially guided	47	25.0%

**Table 5:** Baseline data of explanatory variables

	Explanatory variable		*n*	Percentage
Guide design-derived factors	Number of teeth supporting the surgical guide	6 teeth	4	2.2%
		7 teeth	7	3.8%
		8 teeth	12	6.4%
		9 teeth	13	6.9%
		10 teeth	33	17.6%
		11 teeth	31	16.5%
		12 teeth	53	28.2%
		13 teeth	20	10.7%
		14 teeth	5	2.7%
	Number of anchor pins	0	138	73.4%
		1	33	17.5%
		2	15	8.0%
		3	2	1.1%
	Presence or absence of reinforcement structure	Absence	130	69.1%
		Presence	58	30.9%

**Table 6:** Influence of each factor on error of the placement position (*P* values)

	Angle	Base	Apex
**Missing teeth-derived factors**			
Type of tooth	0.853	0.339	0.729
Type of edentulism	0.915	0.882	0.780
Distance from remaining teeth to placement position	0.019	0.837	0.614
**Implant-derived factors**			
Type of implant	0.274	0.205	0.258
Implant length	0.131	0.071	0.047
Number of implants	0.003	0.031	0.008
Method of guidance	< 0.001	0.023	0.001
**Guide design-derived factors**			
Number of teeth supporting the surgical guide	0.041	0.015	0.007
Number of anchor pins	< 0.001	0.702	0.221
Presence or absence of reinforcement structure	< 0.001	0.608	0.052

### Missing teeth-derived factors

**Fig. 6:**
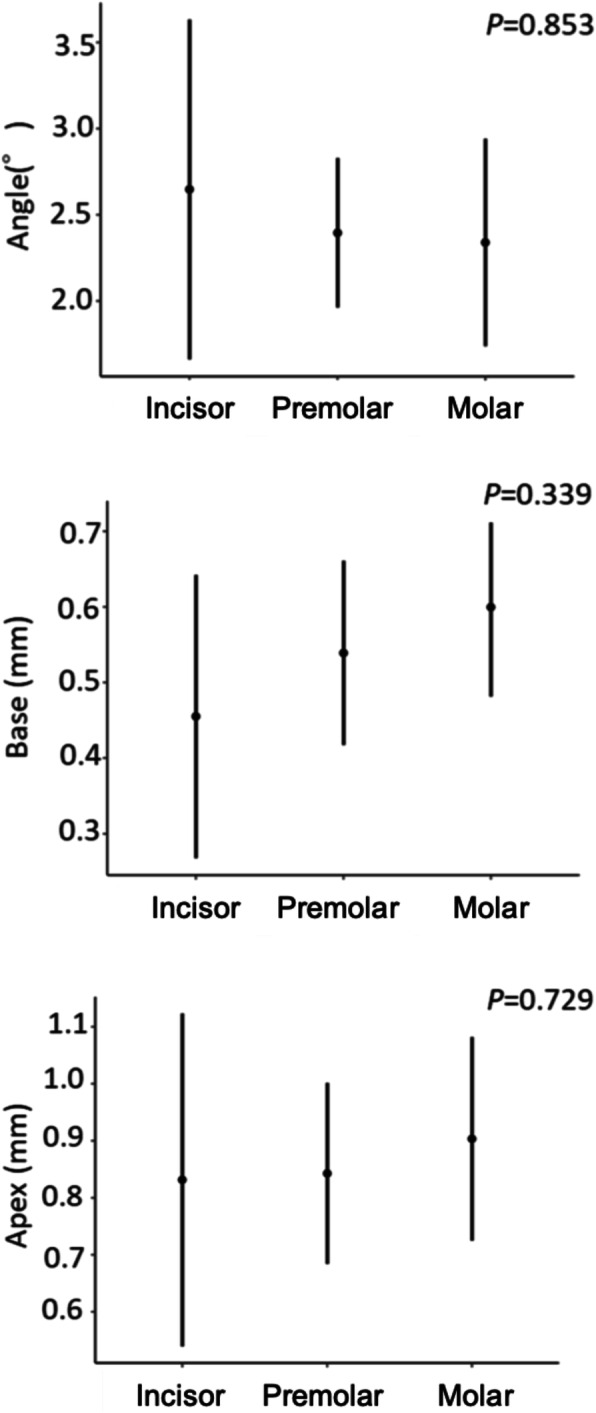
The errors caused by differences in type of tooth. Error bars indicate 95% confidence intervals

**Fig. 7:**
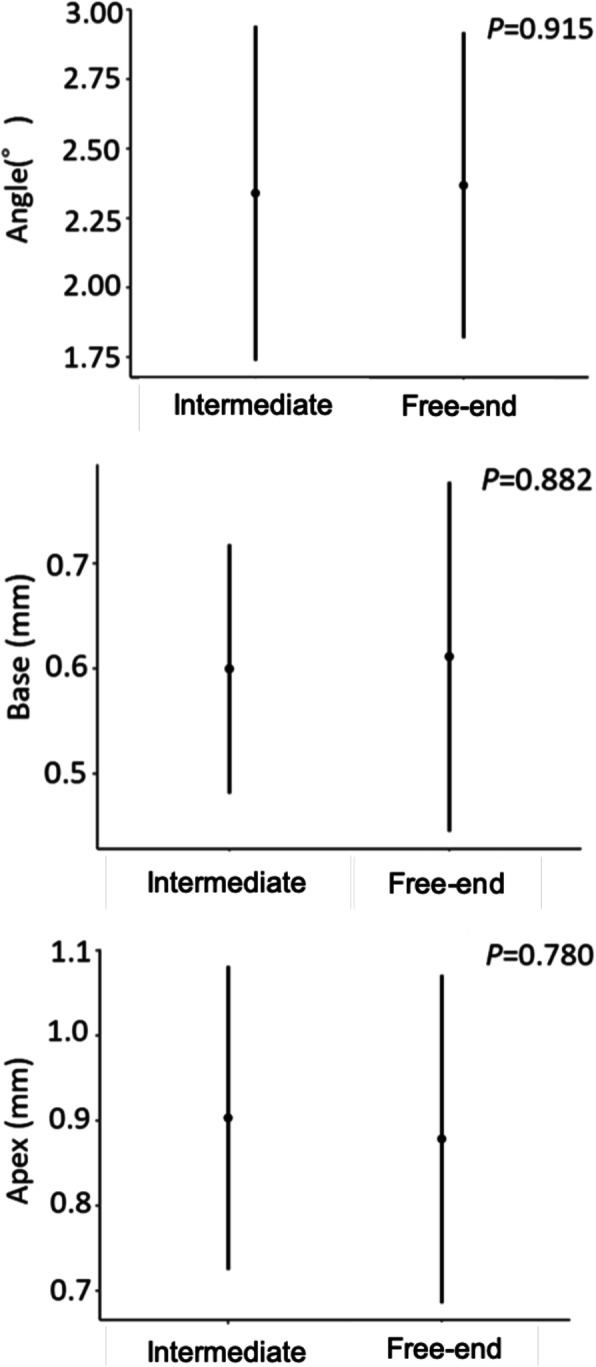
The errors caused by differences in type of edentulism. Error bars indicate 95% confidence intervals

**Fig. 8:**
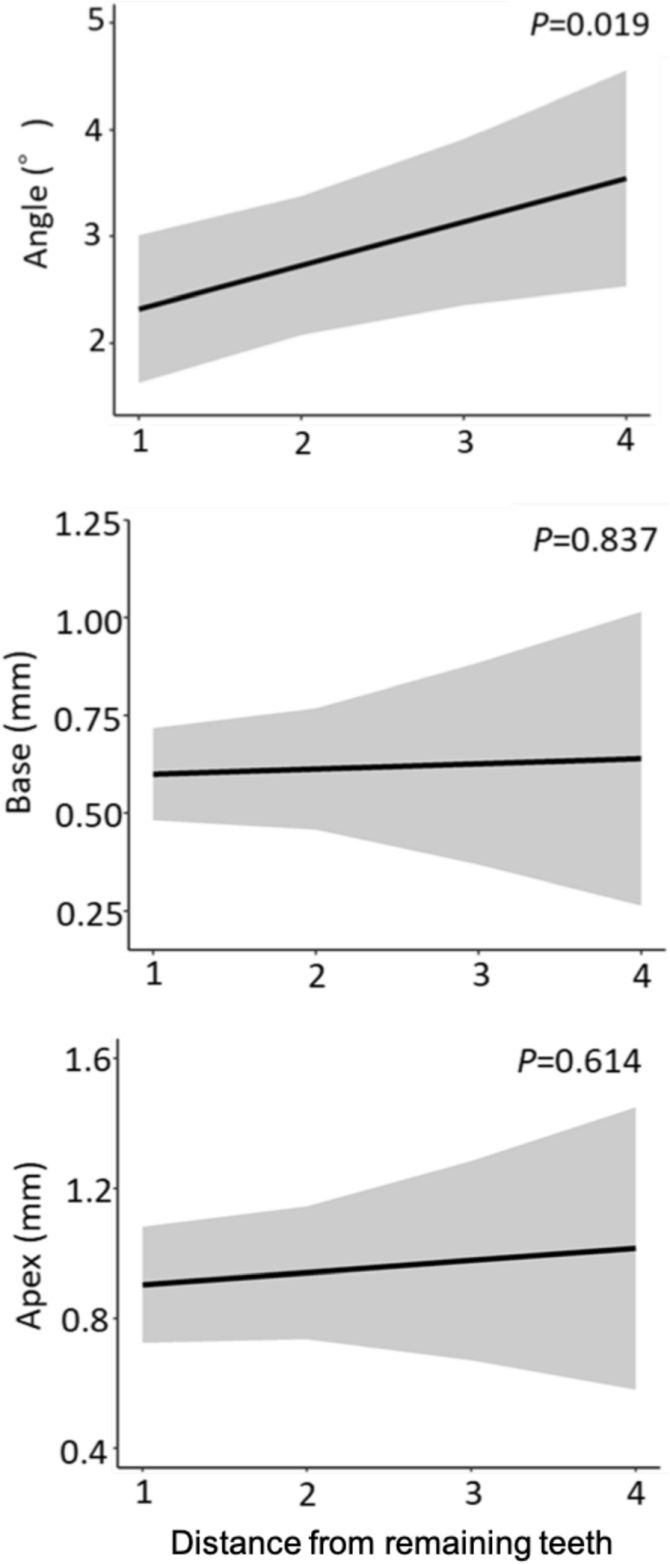
The errors caused by differences in distance from remaining teeth to the placement position. The shaded area shows 95% confidence intervals

There were no significant differences in the errors in the placement position caused by differences in the type of tooth or type of edentulism (Figs. 6 and 7). There was no significant difference between the Base and Apex in the error in the placement position caused by the difference in the distance from the remaining teeth to the placement position; for the Angle, however, the error increased as the distance from the remaining teeth to the placement position increased (*P* = 0.019) (Fig. 8).

### Implant-derived factors

**Fig. 9:**
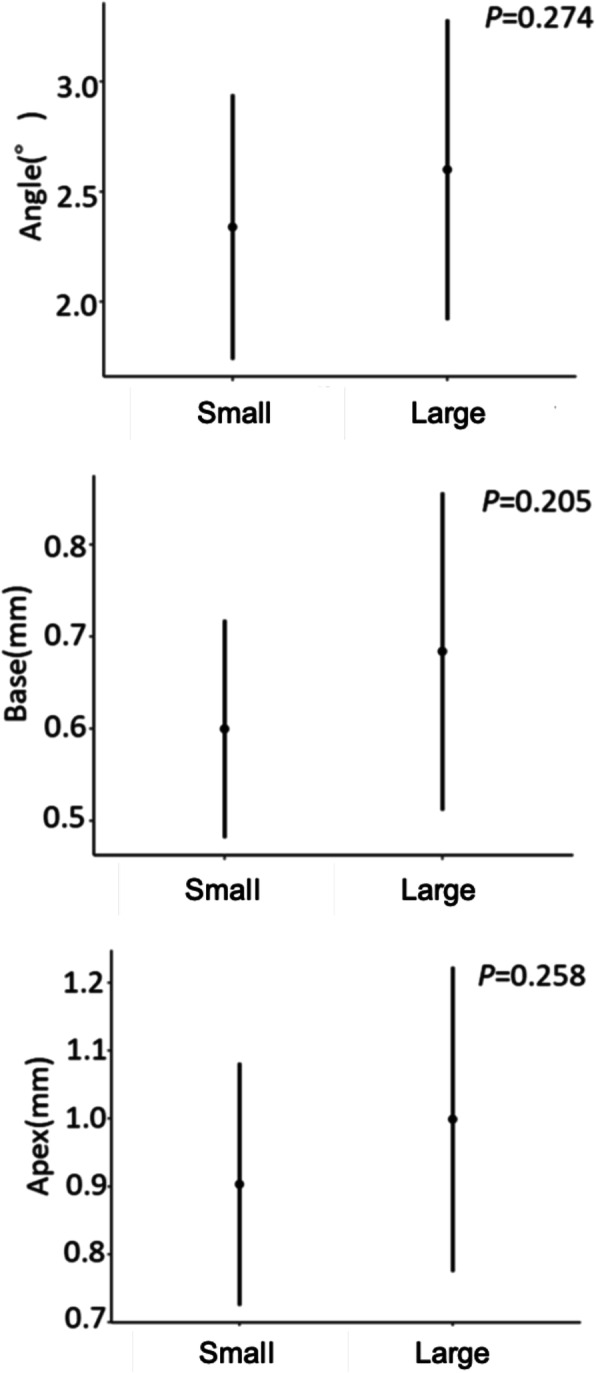
The error caused by the difference in the type of implant with large and small diameter differences between the implant mount and the metal sleeve. Error bars indicate 95% confidence intervals

**Fig. 10:**
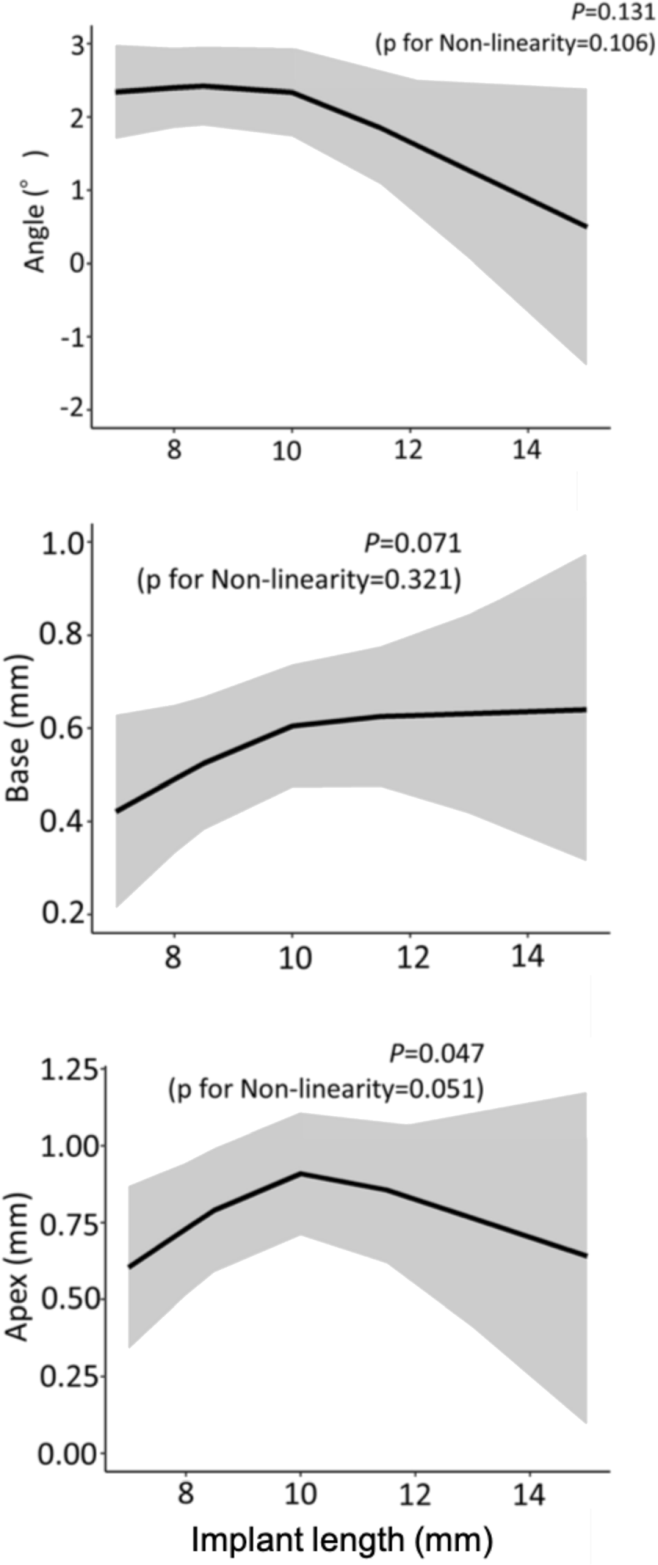
The errors caused by differences in the implant length. The shaded area shows 95% confidence intervals

**Fig. 11:**
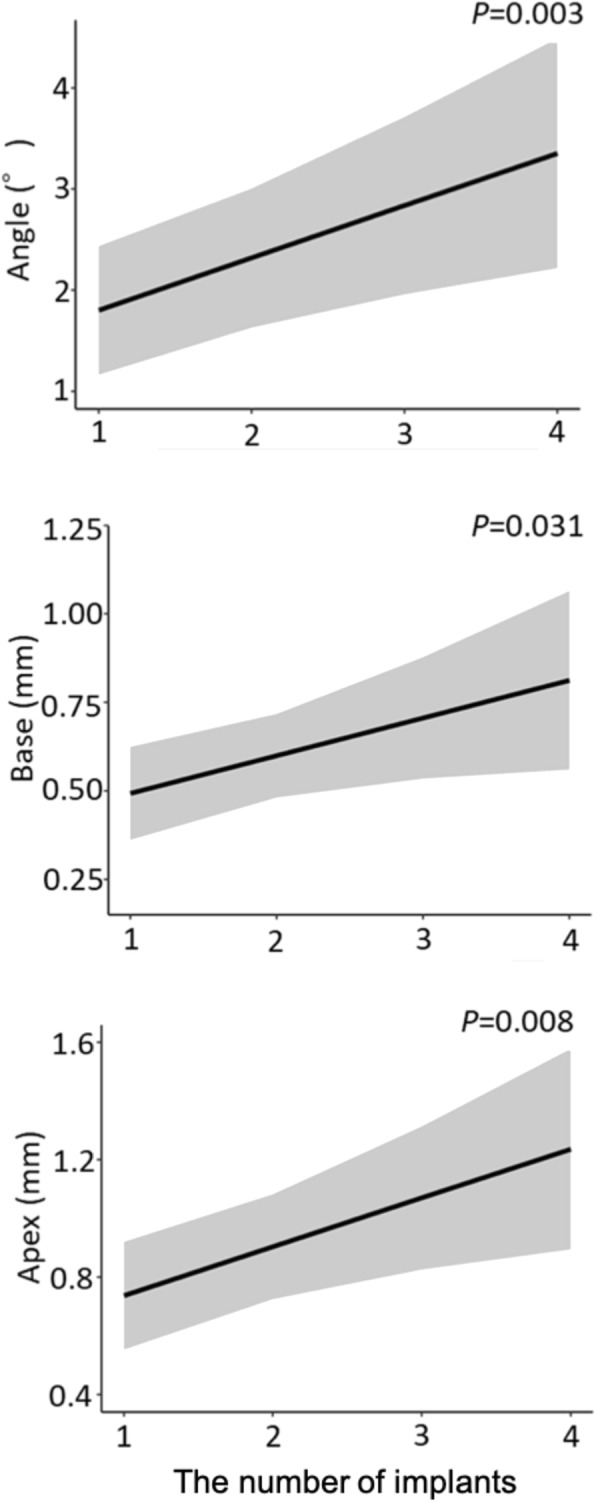
The errors caused by differences in the number of implants. The shaded area shows 95% confidence intervals

**Fig. 12:**
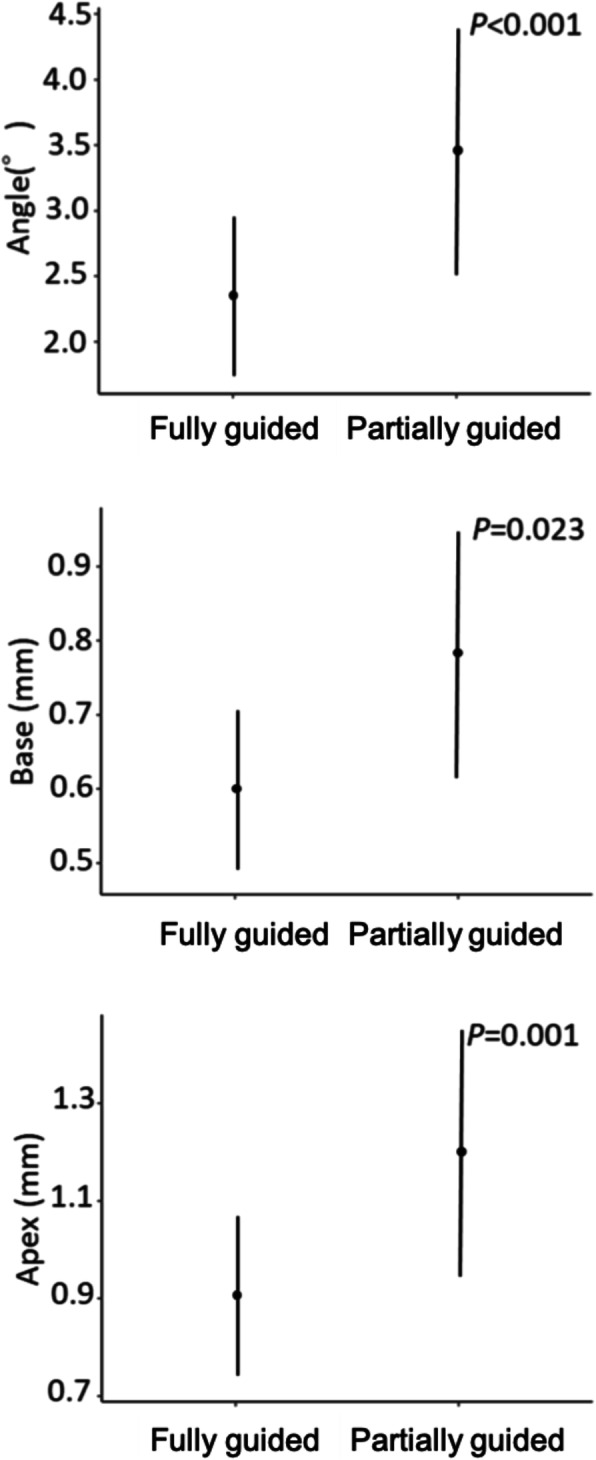
The error caused by differences in the method of guidance. Error bars indicate 95% confidence intervals

The error in the placement position caused by the difference in the type of implant showed no significant difference in all outcomes (Fig. 9). The error of the placement position caused by the difference in the implant length had a significant difference in the Apex, and the error increased as the length increased (*P* = 0.047) (Fig. 10). The error in the placement position caused by the difference in the number of implants per patient significantly increased as the number of implants increased for all parameters: Angle (*P* = 0.003), Base (*P* = 0.031), and Apex (*P* = 0.008) (Fig. 11). The error in the placement position caused by the method of guidance showed a significant difference for all parameters: Angle (*P* <0.001), Base (*P* = 0.023), and Apex (*P* <0.001) (Fig. 12). The partially guided group had larger errors than the fully guided group.

### Guide design-derived factors

**Fig. 13:**
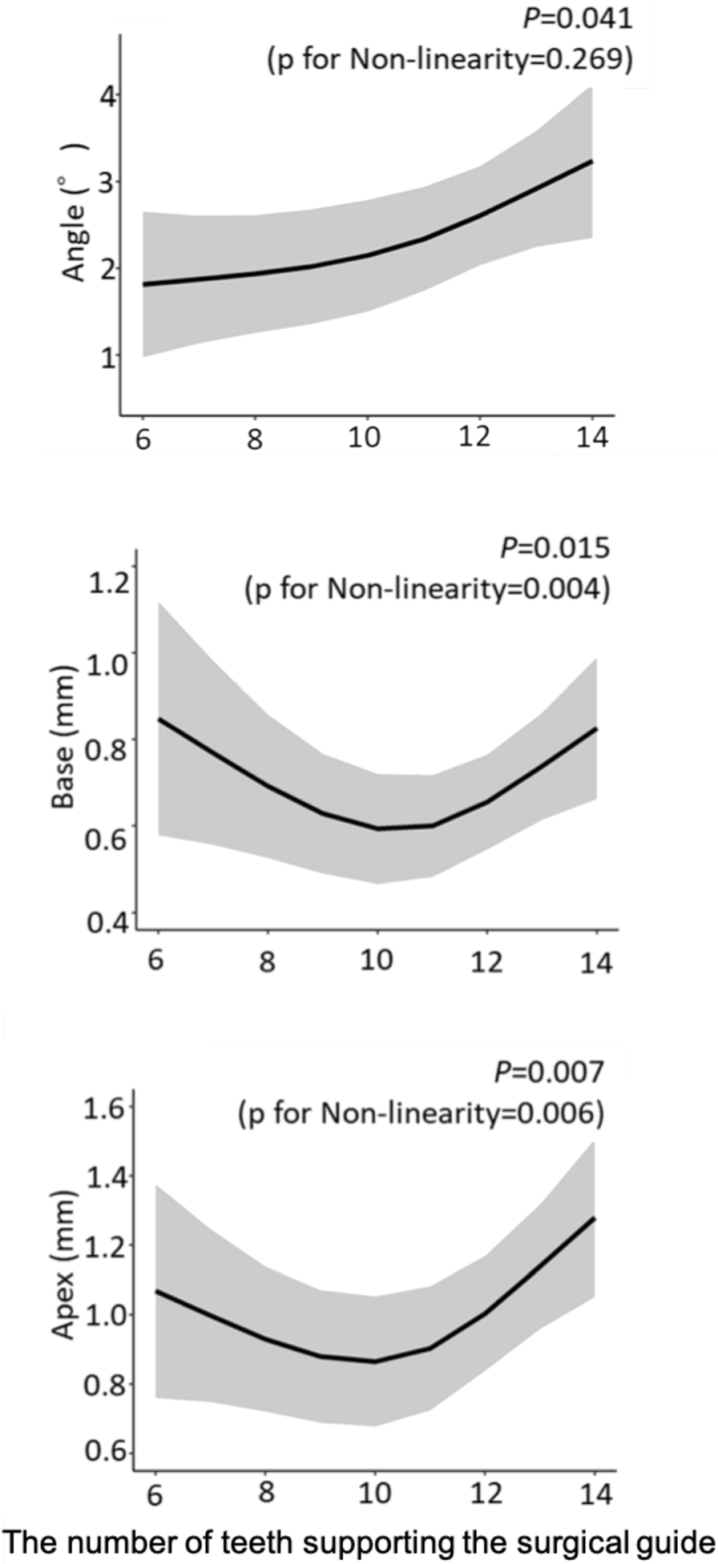
The errors caused by differences in the number of teeth supporting the surgical guide. The shaded area shows 95% confidence intervals

**Fig. 14:**
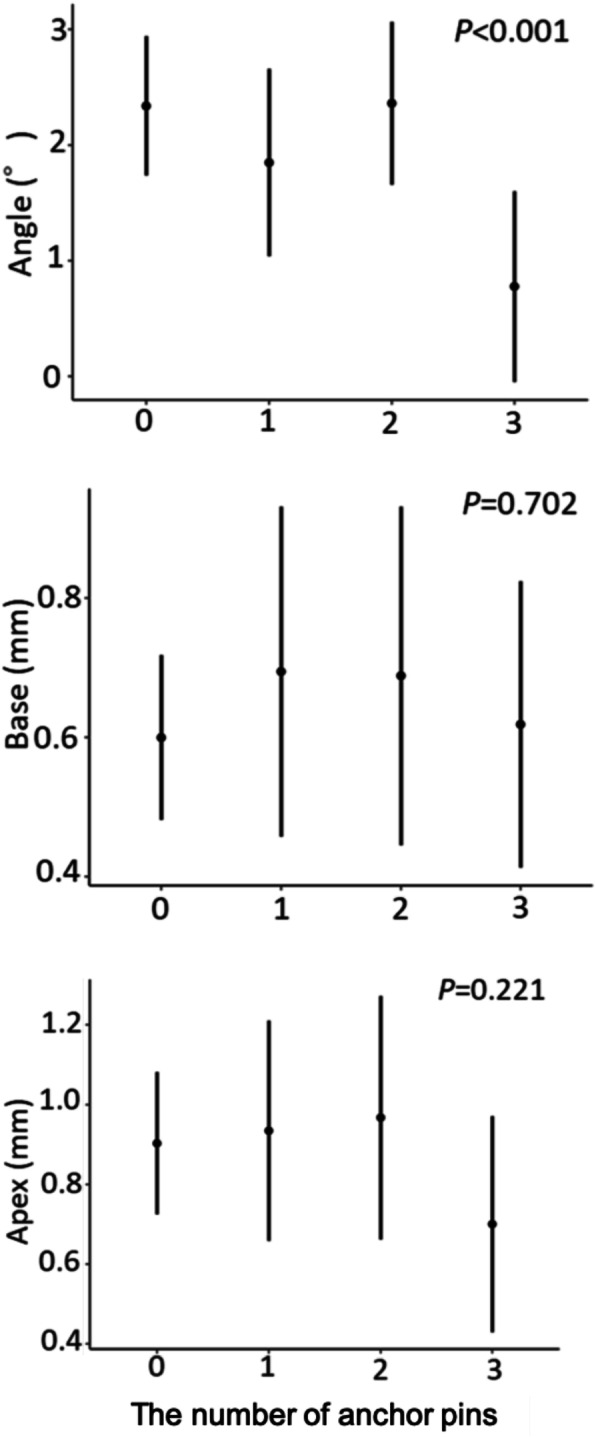
The error caused by the differences in the number of anchor pins. Error bars indicate 95% confidence intervals

**Fig. 15:**
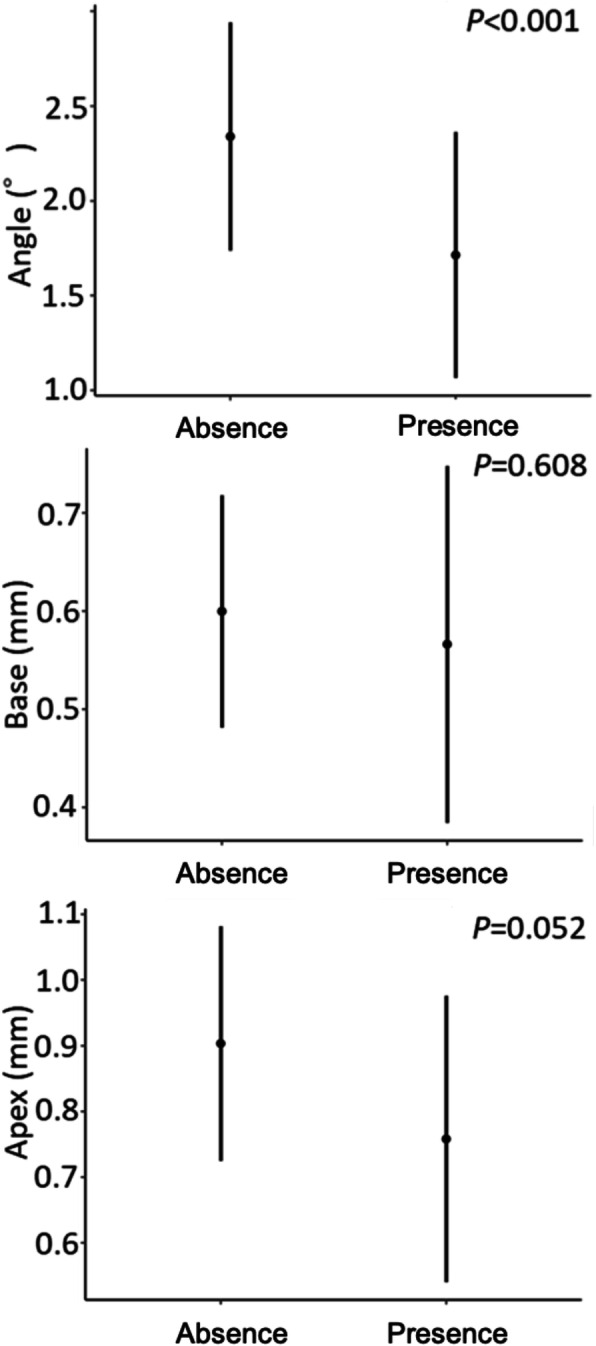
The error caused by the presence or absence of the reinforcement structure. Error bars indicate 95% confidence intervals

The error in the placement position caused by the difference in the number of teeth supporting the surgical guide was significantly different for the Angle (*P* = 0.041), Base (*P* = 0.015), and Apex (*P* = 0.007) (Fig. 13). In the Base and Apex, the error decreased as the number of support teeth increased, and the error became smallest at around 10 teeth; the error then increased inversely as the number of support teeth increased further. The error in the placement position caused by the difference in the number of anchor pins was significantly different for the Angle (*P* < 0.001) (Fig. 14). The error in the placement position tended to decrease as the number of anchor pins increased. Similarly, the error in the placement position caused by the presence or absence of the reinforcement structure was also significantly different for the Angle (*P* < 0.001) (Fig. 15). The error was smaller in the group with reinforcement structure.

## Discussion

In the report by Tahmaseb et al. [1], the error of the placement position was as follows: Angle, 3.5° (95% confidence interval, 3.00–3.96°); Base, 1.2 mm (95% confidence interval, 1.04–1.44 mm); and Apex, 1.4 mm (95% confidence interval, 1.28–1.58 mm). These error values are larger than those in the present study. This is considered to be because the present study is limited to partial edentulism, and since the measurement method of implant validation is different, a simple numerical comparison is not possible.

### Missing teeth-derived factors

Errors in the placement position caused by differences in the type of tooth and type of edentulism did not differ significantly in all measurement outcomes, but this result differs from previous reports. This is probably because Vasak et al. [14] classified the type of tooth into two groups (anterior teeth and molars), and Behneke and Burwinkel, [25] distinguished between the type of edentulism by a single missing tooth versus multiple missing teeth. These studies did not exclude the effects of other confounding factors by statistical processing.

The error in the placement position caused by the difference in the distance from the remaining teeth to the placement position was significantly different only for the Angle. Thus, if the distance from the remaining teeth to the placement position is large, the placement direction is likely to be incorrect even if guided surgery is used because the adjacent tooth serves as an indicator of the placement direction.

Of the three types of missing teeth-derived factors, the only significant outcome was the Angle at the distance from the remaining teeth to the placement position. This result suggests that in guided surgery, the effect of the missing teeth-derived factor on the error of the placement position is smaller than the effects of other factors.

### Implant-derived factors

Errors in the placement position caused by differences in the type of implant were not significantly different in all measurement outcomes. In past reports, a smaller difference in diameter between the sleeve and the drill was associated with a smaller error [17]; however, there is a concern that heat generation may occur by friction between the sleeve and the drill [30]. The difference in diameter between the sleeve and the implant mount of the implant system used in this study was designed to prevent the torque caused by their contact, and the difference tended to be larger in the larger gap group. However, no statistical significance was found.

The error in the placement position caused by the difference in the implant length was significantly different only for the Apex. Therefore, although using guided surgery can reduce errors near the platform, errors at the tip are more likely to occur as the implant lengthens, and more careful planning and surgery are required when using long implants. In past reports, the error was larger for long than short implants [17, 18]; however, the number of implant body samples in these reports is considered too small (8 and 14, respectively). In contrast, some reports have indicated that the implant length does not affect the error of the placement position [26, 31], and no clear conclusion has been obtained. The present study involved as few as 10 implant bodies of ≥ 13 mm; thus, care must be taken in interpreting the results. However, since the total number of samples was 188 and a nonlinear analysis was performed, more reliable results seem to have been obtained.

As the number of implants per patient increased, the error in the placement position significantly increased for all measurement items. This suggests that if the number of implants is large, motion and deflection of the surgical guide occur and the error increases. In past reports [13, 22, 32, 33], the number of implants per subject was about 5; in contrast, this number was 1.71 in the present study. This is because the present study focused only on patients using a method called SmartFusion (Nobel Biocare), in which a surgical guide is produced based on the scan data of a model, and six or more remaining teeth are needed in this method [34]. This might have affected the number of implants. Because the maximum number of implants per patient in this study was four, it is necessary to take into consideration that the results obtained in this study are applicable to patients with few missing teeth.

In addition, the error in the placement position caused by the method of guidance showed a significant difference in all measurement outcomes. The error in the placement position was smaller in the fully than partially guided group. Thus, the accuracy of guided surgery performed with full guidance in the present study was as high as in previous reports [27, 35–39].

### Guide design-derived factors

The errors in the placement position caused by the difference in the number of teeth supporting the surgical guide were significantly different in all measurement outcomes. For the Base and Apex, the error decreased as the number of support teeth increased at around 10 teeth and then increased as the number of support teeth increased. Because the gradient of the graph also changed at around 10 teeth for the Angle, the optimal number of teeth supporting the guide appears to be around 10 teeth. Most studies on the precision of guided surgery have included the mucosal support to their data [40, 41] and few previous studies focusing on tooth-supported guides have examined the number of these supportive teeth. Kholy et al. [42] reported guides supported by 4 teeth were not significantly different from the accuracy of full-arch-supported guides; however, this report does not treat the number of teeth supporting the guide as a continuous variable. In the present study, the influence of the type of surgical guide support was excluded by limiting it tooth-supported guide, and the influence according to the number of supportive teeth was examined in detail. Too much or too little teeth supporting the guide are considered unfavorable to balance fit and stability. However, further research is needed to confirm this.

The error in the placement position caused by the number of anchor pins was significantly different for the Angle. The error in the placement position tended to decrease as the number of anchor pins increased. Setting the anchor pin seems to be effective in preventing deviation of the angle, because operators easily fix the surgical guide during surgery [31].

The error in the placement position caused by the presence or absence of the reinforcement structure showed a significant difference for the Angle. This suggests that setting the reinforcement structure was effective in preventing deviation of the angle. In particular, because errors of the Apex tend to be smaller with than without a reinforcement structure, the errors at the tip of the implant can likely be reduced by suppressing the deflection of the surgical guide by the reinforcement structures. Van Assche et al. [16] and Tatakis et al. [43] also noted that deflection or fracture of the surgical guide is a factor that causes an error in the placement position, and this can be interpreted as a supportive result.

Based on past studies [14–20, 23–28] and clinical experience, this study has enumerated the factors that seem to affect the error of the placement position, including the design of the surgical guide. By correcting the confounding of each factor using the multivariate analysis, the magnitude of the influence of each factor on the placement position error was calculated. Although there are many other factors that cause errors in addition to the factors listed in this study, the number of samples is limited, so no further explanatory factors were added to maintain the power of statistics. Certainly, the factors related to the so many surgeons are not included in the explanatory variables, so it is undeniable that differences in operators may affect the results. However, it is generally reported that the guided surgery is not easily affected by the difference in the experience value of the surgeon [44], and the effect is considered to be limited.

The above findings indicate that the error in the placement position caused by the missing teeth-derived factor does not differ significantly in most of the measurement outcomes, while the error in the placement position caused by the implant-derived factor and the guide design-derived factor significantly differs in many measurement outcomes. As a result, factors that can be controlled by the operator have a greater effect on the error of the placement position than the oral condition of each patient. Above all, these findings suggest that the smaller errors could be present by performing guided surgery with full guidance and devising the design of the surgical guide. However, because the results include selection bias, research involving a more advanced study design is necessary, such as performing random assignment to obtain a higher level of evidence.

## Conclusion

In this study, we examined the factors that affect errors of implant placement position in implant guided surgery using a multivariate analysis. Our findings clearly indicate that the factors that can be controlled by the operator, such as implant-derived factors and guide design-derived factors, have a greater influence on errors of the placement position than do missing teeth-derived factors. In particular, this study clarified that the design of the surgical guide, such as the number of teeth supporting the surgical guide, the setting of the anchor pin, and the reinforcement structure, influences errors of implant placement position during guided surgery.

## Data Availability

The datasets used and/or analyzed during the current study are available from the corresponding author on reasonable request.

## Abbreviations

CBCT: Cone-beam computed tomography
ICC: Intraclass correlation coefficients
CI: Confidence interval
